# A broadly reactive ultralong bovine antibody that can determine the integrity of foot-and-mouth disease virus capsids

**DOI:** 10.1099/jgv.0.002032

**Published:** 2024-10-18

**Authors:** John D. Clarke, Helen M.E. Duyvesteyn, Eva Perez-Martin, Undīne Latišenko, Claudine Porta, Kathleen V. Humphreys, Abigail L. Hay, Jingshan Ren, Elizabeth E. Fry, Erwin van den Born, Bryan Charleston, Marie Bonnet-Di Placido, Raymond J. Owens, David I. Stuart, John A. Hammond

**Affiliations:** 1 The Division of Structural Biology, Nuffield Department of Medicine, The Centre for Human Genetics, University of Oxford, Oxford, OX3 7BN, UK; 2 The Pirbright Institute, Woking, GU24 0NF, UK; 3 Diamond Light Source, Didcot, OX11 0DE, UK; 4 MSD Animal Health, 5831 AN Boxmeer, Netherlands; 5 Structural Biology, The Rosalind Franklin Institute, Didcot, OX11 0QX, UK; 6 Chinese Academy of Medical Science (CAMS) Oxford Institute (COI), University of Oxford, Oxford, OX3 7BN, UK

**Keywords:** FMDV, pan-specific, single particle analysis, ultralong CDR antibody, vaccine quality assurance

## Abstract

Foot-and-mouth disease vaccination using inactivated virus is suboptimal, as the icosahedral viral capsids often disassemble into antigenically distinct pentameric units during long-term storage, or exposure to elevated temperature or lowered pH, and thus raise a response that is no longer protective. Furthermore, as foot-and-mouth disease virus (FMDV)’s seven serotypes are antigenically diverse, cross-protection from a single serotype vaccine is limited, and most existing mouse and bovine antibodies and camelid single-domain heavy chain-only antibodies are serotype-specific. For quality control purposes, there is a real need for pan-serotype antibodies that clearly distinguish between pentamer (12S) and protective intact FMDV capsid. To date, few cross-serotype bovine-derived antibodies have been reported in the literature. We identify a bovine antibody with an ultralong CDR-H3, Ab117, whose structural analysis reveals that it binds to a deep, hydrophobic pocket on the interior surface of the capsid via the CDR-H3. Main-chain and hydrophobic interactions provide broad serotype specificity. ELISA analysis confirms that Ab117 is a novel pan-serotype and conformational epitope-specific 12S reagent, suitable for assessing capsid integrity.

**Significance as a Bioresource to the Community**

Significant viral capsid structural diversity both between and within foot-and-mouth disease virus (FMDV) serotypes hampers research efforts. Topotype-matched specific reagents are required for accurate detection and distinction of both intact FMDV capsids and dissociated FMDV pentameric units. Given that vaccination efficacy is linked with capsid integrity and that FMDV capsids present vast structural diversity, access to a suite of reagents specific for either intact or dissociated particles across the varied FMDV topotypes is of great importance. This study provides Ab117, an ultralong bovine antibody that binds a deep conformational epitope at the interior surface of 12S particles and that additionally demonstrates broad pan-serotype specific activity with a greater sensitivity and specificity for 12S particles than current reagents. Ab117 is therefore useful as a broadly active 12S detection reagent, contributing a valuable tool to the existing suite of reagents used in FMDV capsid quality assurance. Ab117 enables reliable, clear assessment of FMDV capsid integrity likely across all seven FMDV serotypes.

## Data Summary

The authors confirm that all supporting data, code and protocols have been provided in the article or through supplementary data files. The amino acid sequence of Ab117 is provided in the supplementary material. EM map and model for the SAT2/ZIM/7/83 12S pentamer in complex with Fab117 knob mini-domains are deposited in the wwPDB and EMDB under accession codes PDB: 9G6V and EMDB: EMD-51105.

## Introduction

Foot-and-mouth disease (FMD) is a highly contagious viral infection principally infecting cloven-hoofed ruminants, causing severe pain, debilitation, infertility and irrecoverable losses to milk and meat production. There are no approved therapeutics for infection, and current vaccines are restricted to nations where FMD is endemic (though emergency stocks are maintained in non-endemic nations) due to the inability to distinguish vaccinated from infected animals. Outbreaks have historically been devastating, e.g. the 2001 UK outbreak resulted in the culling of some 6.5 million animals, costing in excess of £8 billion [1,2], whilst in endemic countries, the annual cost is estimated as ca. >1.5 billion USD [3].

The causal agent of FMD, foot-and-mouth disease virus (FMDV), is a non-enveloped picornavirus with an 8.5 kb positive-sense RNA genome. Due to the high mutational frequency of the error-prone FMDV RNA polymerase [4,6], FMDV is highly antigenically diverse. FMDV is grouped into six serotypes [7,9] (a seventh serotype, C, has been eradicated) and subdivided further into many antigenically distinct topotypes/subtypes [10].

During replication, structural proteins are translated as a polyprotein precursor P1, which folds and is then cleaved by the viral 3C protease to form a protomer structural unit of VP0, VP1 and VP3. It is assumed that five copies of this protomer self-assemble into 12S pentameric units [11], which then further assemble around the genome to form the full icosahedral capsid. Since interactions between these pentameric units are relatively weak and involve a number of histidine residues with a pKa around 6.9 [12], the capsids are highly sensitive to temperature, ion concentration and pH (below 7.0) [13]. Following assembly and genome encapsidation, capsids mature via an autocatalytic process separating VP0 into VP4 and VP2, whose termini re-arrange to form an internal protein network providing capsid stability.

Currently, vaccination utilizes inactivated FMDV [2], which is suboptimal due to the necessity of high containment for production, indistinguishability from live virus and instability of the antigen. Next-generation vaccines made up of recombinantly expressed and self-assembled virus-like particles (VLPs) with engineered stabilizing mutations [14,15] should be more straightforward to produce/disseminate, make vaccinated animals distinct from those infected and overcome stability/shelf-life issues [14]. Given that pentamers and intact capsids are antigenically distinct, capsid integrity is an essential measure for vaccine development and quality control (QC). Several anti-/nano-bodies that distinguish pentamers from intact capsids are available, supporting ELISA-based QC assay [16]. However, due to inter- and even intra-serotype antigenic variation, a suite of strain-matched antibodies is required. For several topotypes, there are no antibody or single-domain heavy chain-only antibody (VHH) pairs that can offer such QC.

Compared to those from other species that have been studied, many cattle antibodies have very long CDR-H3's (third heavy-chain complementary-determining region), including a minority that is ultralong (up to 71 residues). These so-called ultralong CDR-H3 antibodies project the CDR-H3 from the usual paratope region as an extended beta-hairpin with a folded ‘knob’ mini-domain at its apical tip [17,23], stabilized by a network of disulphide bonds [19]. This unusual structure creates the potential to recognize epitopes that would otherwise be inaccessible.

In this study, we identify and characterize a novel cattle ultralong antibody, Ab117, that recognizes a non-linear epitope on disrupted VLPs and non-intact viral capsids. We show by cryogenic electron microscopy (cryo-EM) that Ab117 binds to the interior surface of serotype SAT2/ZIM/7/83 FMDV-dissociated pentamers. The 3.2 Å resolution structure shows the binding footprint, revealing that all contacts are with the ultralong CDR-H3, which engages an epitope on the interior surface of the pentamer abutting the protomer quasi-threefold axis, a site inaccessible in the intact capsid. Ab117 binds as if contributing to the tertiary structure of VP2, via mostly main-chain and hydrophobic interactions with a tryptophan sidechain on the very tip of the CDR-H3 mini-domain buried deep into a pocket at the pseudo-threefold axis, locking the antibody in place. Residues involved in the interactions, especially those lining the tryptophan-binding pocket, are highly conserved across serotypes and, together with the preponderance for main-chain interactions, give the antibody broad specificity.

## Methods

### Antibody sequence discovery

The antigens used to vaccinate calves were inactivated FMDV O_1_/MAN/TUR/69 (O1M), A_22_/IRQ/24/64 (A22), Asia1/SHA/ISR/89 (A1S) and unpurified baculovirus-produced SAT2/SAU/6/2000 (S2S) VLPs prepared as previously described [15]. Antigens were supplied as crude PEG precipitates from culture supernatants by MSD Animal Health (Boxmeer, The Netherlands) and purified by dilution of the PEG precipitate 1/8-1/12 in HEPES 50 mM NaCl 200 mM pH 8.0 (HN0.2), followed by clarification at 10 000 ***g*** for 20 min at 12 °C. The resulting PEG pellets were extracted with 100 mM HEPES and 500 mM MgCl_2_ pH 8.0 (H1M5). Virus from both PEG supernatant and pellet extracts was subsequently pelleted over a 30% sucrose cushion at 145 600 ***g*** for 2 h 30 min at 12 °C. The resulting pellets were resuspended into 1 ml of buffer HN0.2. In the case of the PEG pellet washes in H1M5, the sucrose cushion pellet resuspendates were treated for 10 min on ice with 0.2 mg ml^−1^ RNAse A and detergent: 0.5% IGEPAL (Sigma) in the case of O1M, 1% IGEPAL in the case of A1S and 0.1% Triton X-100 (Sigma) in the case of A22. Following clarification at 10 000 ***g*** for 10 min at 4 °C, all sucrose cushion pellet resuspendates were loaded onto 10–50% sucrose gradients prepared in buffer HN0.2 and centrifuged at 154 300 ***g*** for 3 h 20 min at 12 °C. The band of 146S virus particles was harvested. To increase purity, the target sucrose fractions were desalted into buffer HN0.2 using a 7 kDa molecular weight cut off (MWCO) filter (Zeba, Thermo Fisher Scientific) and loaded onto a second 10–50% sucrose gradient in buffer HN0.2. The band of 146S particles was again harvested, prior to quantification by ELISA and formulation for immunization.

Quantification prior to vaccination by VHH ELISA was as previously described [16] with minor modifications. Briefly, high-binding polystyrene 96-well plates (Thermo Fisher Scientific, Waltham, MA, USA) were coated with a serotype-specific anti-FMDV VHH [Wageningen Bioveterinary Research (WBVR), Lelystad, The Netherlands; Table S1, available in the online version of this article] at 0.5 µg ml^−1^ in coating buffer (50 mM, carbonate/bicarbonate buffer, pH 9.6) and incubated overnight at 4 °C. All subsequent incubation steps were performed for 1 h at room temperature, with orbital shaking (180 r.p.m.), and all washing steps were performed with 200 µl of wash buffer (PBS 0.05% Tween 20). Antigens and antibodies were diluted in PBS 1% skimmed milk and 0.05% Tween 20. After VHH coating and washing, the plates were incubated with 50 µl of serial threefold dilutions of purified classic inactivated antigens or unpurified VLPs (in the case of S2S) and inactivated FMDV antigen standards, either intact or disrupted by heating at 56 °C for 15 min [14]. Later, 100 µl of biotinylated anti-FMDV VHH (Wageningen Bioveterinary Research) at 0.2 µg ml^−1^ were added, followed by 100 µl of streptavidin-HRP (1 : 10 000; Thermo Fisher Scientific). Bound peroxidase conjugate was subsequently detected with 100 µl of tetramethylbenzidine (TMB) (Invitrogen, Thermo Fisher Scientific), and the reaction was stopped with 100 µl of stop solution (Invitrogen, Thermo Fisher Scientific) when the appropriate colour was reached. Absorbance was read at 450 nm using a Tecan Infinite M200 microplate reader (Tecan Group Ltd., Männedorf, Switzerland).

Six male 5- to 6-month-old Holstein-Friesian calves (*Bos taurus*) obtained from the Centre for Dairy Research (University of Reading, UK) were divided into a group of four (vaccinated group) and a nonvaccinated control group of two. Each calf in the vaccinated group received 10 µg of O1M for the prime vaccination at day 0 and then was sequentially vaccinated with 6–8 µg of O1M, 10 µg of A22, 10 µg of A1S purified classic antigens and 10 µg of unpurified SAT2/SAU/6/200 VLPs on days 21, 42, 63 and 84, respectively, after primary vaccination. All vaccines were administered intramuscularly above the right prescapular lymph node and formulated in Montanide ISA 206/HEPES-KCl adjuvant (MSD Animal Health). The control calves were administered an adjuvant-only vaccine at each vaccination timepoint. Heparinized blood samples were taken from each calf from both groups at multiple timepoints following the primary and boost vaccinations. All experiments were approved by The Pirbright Institute’s ethical review process and followed national guidelines on animal use.

Cattle PBMCs were isolated from heparinized blood samples as described by Valdez *et al.* [24], with a few modifications. Briefly, blood samples were diluted 1 : 2 with Dulbecco’s PBS (DPBS) without calcium and magnesium (Gibco, Thermo Fisher Scientific) and added to SepMate PBMC isolation tubes (Stemcell Technologies, Vancouver, BC, Canada) containing Histopaque at 1.083 g ml^−1^ (Sigma-Aldrich, Merck, Darmstadt, Germany). Tubes were centrifuged for 30 min at 1200 ***g***. After centrifugation, diluted plasma from the top layer was aspirated and stored at −80 °C until further use, and the mononuclear cell layer was harvested and treated with ACK buffer (Gibco) to lyse the remaining red blood cells. The recovered mononuclear cells were washed twice with ice-cold DPBS, and cell numbers were obtained using the TC10 Automated Cell Counter (Bio-Rad, Hercules, CA, USA). Cells were resuspended in Horse Serum 10% DMSO (Sigma-Aldrich) for cryopreservation [24]. PBMCs and vaccinia virus (VV VLPs were sent to AbCellera (Vancouver, Canada) for FMDV-specific IgG B cell isolation and paired antibody heavy and light chain sequencing.

### Production of recombinant antibodies

Synthetic genes encoding the variable-diversity-joining heavy chain gene segment rearrangement or variable-joining light chain gene segment rearrangement coding fragments of FMDV-specific antibodies were purchased from IDT Technology as gBlocks and directionally cloned into the pNeoSec-BovFc-IgG1 and pNeoSec-BovLC-λ expression vectors for recombinant IgG1 production, or pOPINBOVH and pOPINBOVL expression vectors [25] for recombinant fragment antibody (Fab) production using InFusion ClonExpress II One Step Cloning Kit (Vazyme). Each vector was transformed into chemically competent *Escherichia coli* Stellar cells (Takara Bio Inc.) according to the manufacturer’s protocol. For each transformation, two clones were sequenced to confirm successful cloning. Recombinant IgG1 ‘Ab117’, or fragment antibody ‘Fab117’, was produced by transient co-transfection of Expi293 cells [26] using corresponding pairs of pNeoSec-BovFc-IgG1 and pNeoSec-BovLC-λ vectors, or pOPINBOVH and pOPINBOVL vectors respectively. Ab117 was purified from culture supernatants by affinity chromatography implemented using Protein G (5 ml columns) on an ÄKTA Pure HPLC system (Cytiva) and buffer exchanged into DPBS. Fab117 was purified from culture supernatants by automated tandem immobilised metal ion affinity chromatography coupled with size exclusion chromatography (IMAC-SEC) implemented on an ÄKTA Xpress HPLC system [27] (GE Healthcare) equipped with a 5 ml HisTrap column (Cytiva) and a HiLoad Superdex200 16/60 column (Cytiva). Eluted fractions were characterized by SDS-PAGE, and those of interest were spin-concentrated using Amicon Ultracel-30 centrifugal filters (Merck, Darmstadt, Germany). IgG1 or Fab concentration was determined from the absorbance at 280 nm.

### Production of virus-like particles by vaccinia virus expression system

VLPs were produced by co-infection of HEK293T cells using recombinant vaccinia viruses (VVs) as previously described [14]. Briefly, recombinant VVs containing either an FMDV P1-2A-3C cassette under the command of a T7 promoter or expressing the T7 RNA polymerase from the p7.5 promoter (vTF7.3) were co-infected into HEK293T cells with multiplicities of infection of 10 and 5, respectively. Engineered FMDV cassettes included serotypes SAT2/ZIM/7/83 with S2093Y (VP residue numbers are prefixed with a numeral corresponding to their VP and hence S2093Y is the serine–tyrosine substitution made at residue 93 of VP2) mutation [28], O_1_/MAN/TUR/69 and Asia1/SHA/ISR/89 both with a S2093C VP2 mutation [29,30], and A22/IRQ/24/64 with a H2093C VP2 mutation [14]. Infected cells were pelleted 24 h later and lysed using 0.5% (v/v) IGEPAL (Sigma). Cellular debris was pelleted and clarified in 50% (v/v) chloroform. VLPs were pelleted using a 30% sucrose cushion ultracentrifugation and resuspended in 40 mM sodium phosphate and 150 mM sodium chloride. VLPs were then further clarified with 0.1% (v/v) IGEPAL and 0.2 mg ml^−1^ RNAse A and fractionated by ultracentrifugation using a 15–45% sucrose density gradient. Collected fractions were characterized by SDS-PAGE and quantified by Qubit (Thermo Fisher Scientific), whilst VLP presence was confirmed by VHH ELISA. Immediately prior to use in single-particle electron cryomicroscopy experiments, VLPs were spin-concentrated using Amicon Ultracel-100 centrifugal filters.

### Single-particle analysis by cryogenic electron microscopy

The Fab117-SAT2 pentamer complex was prepared allowing for a 3× molar excess of Fab117 per anticipated epitope at a final concentration of 1 mg ml^−1^ and incubated for 10 min without the need for further purification. Quantifoil R2/1 Copper 300 Mesh EM grids were glow discharged for 30 s on high setting (Harrick Plasma) before 3 µl of the complex was applied, blotted and vitrified in liquid ethane by plunge freezing using a Vitrobot Mark IV (Thermo Fisher). Electron cryomicroscopy data were collected on a Titan Krios G3i equipped with a Gatan K2 detector and energy filter at 9.09 e^-^/Å^2^/s with a 20 eV slit over a defocus range of −2.8 µm to −1.4 µm in 0.2 µm increments. The resultant pixel size was 1.054 Å/px, corresponding to a nominal magnification of 130 kX. Micrographs were processed using CryoSPARC [31] v3.3.2 (Structura Biotechnology) implemented in the Biomedical Research Computing facility within the Nuffield Department of Medicine. From 13 327 movies, 493 093 particles were picked (two rounds of blob picking, followed by two rounds of template picking), further sorted in 2 rounds of 2D classifications before use in unbiased *ab initio* 3D reconstruction. A total of 110 210 particles were used in the refinement of the final cryo-EM density map and resolution estimated by gold standard Fourier shell correlation (FSC) in CryoSPARC v3.3.2 [31] (Table 1).

**Table 1.** Single-particle analysis. Data collection, structure determination and refinement statistics

Data collection		Model refinement	
Accelerating voltage (kV)	300	Map resolution (GSFSC=0.314; Å)	3.2
C2 aperture (µm)	70	D FSC model (0.5)	3.3
Energy filter width (eV)	20	CC_1/2_ (Mask)	0.82
Calibrated pixel size (Å/px)	1.054	Sharpening B-factor (Å^2^)	51
Nominal magnification (kX)	130	No. atoms	21 815
Dose rate (e^-^/Å^2^/s)	9.1	B-factors (Å^2^) min/max/mean	14/185/81
EER fractions	40	Root mean squared deviations	-
Total dose (e^-^/Å^2^)	40.0	Bond lengths (Å)	0.003
Exposure (s)	4.4	Bond angles (°)	0.4
Defocus range (µm)	−2.8 : −1.4	Clash score	5.4
Movies	13 327	Ramachandran	-
Particles (final)	110 210	Favoured/allowed/outliers (%)	95.3/4.7/0.0
Symmetry imposed	C5	Rotamer outlier (%)	0.68

### Protein structural prediction using AlphaFold

The structure of Fab117 was predicted using the multimer mode of AlphaFold v2.0 [32]. The VH and CH1 domains of the heavy chain (HC), and the light chain (LC) were used as inputs. The prediction was run on 10 January 2023 using default settings. The structure of the VLP pentamer and the ordered portion of CDR-H3 were rigid-body fitted into the cryo-EM density, rebuilt and refined through iterations of Coot [33] and Phenix [34]. Data processing and refinement statistics are summarized in Table 1.

### ELISA for detection of 146S and 12S particles using Ab117

To investigate 146S and 12S detection with Ab117, three separate ELISAs were set up.

#### Homologous single-domain heavy-chain/antibody ELISA

Capsid integrity was assessed by double sandwich ELISA. High-binding 96-well plates (Thermo Fisher) were coated overnight with a serotype-specific capture VHH (produced by WBVR or The Immunological Toolbox [35]; Table S1) or with Ab117 diluted to 0.5 mg ml^−1^ in 50 mM bicarbonate coating buffer (0.035 M sodium hydrogen carbonate, 0.01 M sodium carbonate, pH 9.6) and washed thrice with wash buffer [0.05% (v/v) Tween20 in 1× PBS] before capsid samples (and appropriate standards) diluted in wash buffer supplemented with 1% (w/v) skimmed milk powder were added to the plate and incubated at room temperature for 1 h. Following subsequent washes, biotinylated serotype-specific detection VHH/antibody (75S or 12S accordingly; WBVR, The Immunological Toolbox [35]; Table S1) was diluted to 0.2 mg ml^−1^ in milk-supplemented wash buffer, applied to the plate for 1 h at room temperature and washed away as above. Pierce High Sensitivity Streptavidin-HRP (Invitrogen) was diluted 1 : 10 000 in milk-supplemented wash buffer, added to the plate for 1 h and subsequently washed away. Signal was obtained by the addition of TMB Substrate Solution (Invitrogen) and ELISA Stop Solution (Invitrogen), and absorbance was read by an Infinite 200 Plate Reader (Tecan) at 450 nm. The observed absorbance values were normalized for background and sample concentration and compared against the untreated and thermally treated (heated to 56 °C for 15 min [14]) standards of the same serotype. All steps were performed with orbital agitation at 150 r.p.m.

#### Universal sandwich integrin ELISA for 146S detection

96-well MaxiSorp plates (Nunc) were coated with 100 µl per well of 0.25 µg ml^−1^ integrin α_v_β_6_ [36] diluted in coating buffer (50 mM Tris, 150 mM NaCl, 2 mM CaCl_2_, 1 mM MgCl_2_ and pH 7.6) and incubated overnight at 4 °C. The plates were washed three times with 300 µl per well wash buffer (0.01 M PBS, 0.15% polysorbate 20, 0.64 M NaCl and 0.0027 M KCl). After 1 h of blocking at 37 °C with 200 µl per well blocking buffer (0.04 M PBS+2% BSA), the plates were washed three times with 300 µl per well wash buffer. The antigen samples, prediluted to ~1 µg ml^−1^ in coating buffer +2% BSA (dilution buffer), were added to the plate and incubated for 1 h at 37 °C. After subsequent wash, Ab117 prediluted to 1 µg ml^−1^ in dilution buffer was added to the plate, 100 µl per well. After another 1 h incubation at 37 °C and subsequent washing, an anti-Ruminant-PO conjugate in dilution buffer was added, 100 µl per well. After 1 h incubation at 37 °C, plates were washed three times with 300 µl per well wash buffer. Substrate (3,3′,5,5′-TMB) in urea hydrogen peroxide buffer (composition and manufacturer) was added, 100 µl/well, and incubated at room temperature in the dark for at least 15 min. The substrate reaction was stopped by adding 50 µl per well 2 M (4 N) sulphuric acid. The optical density at 450 nm (OD450) value of each well was read using a Tecan Sunrise spectrophotometer.

#### Universal direct ELISA for 12s detection

96-well MaxiSorp plates (Nunc) were coated with 100 µl per well of heat-treated (1 h, 56 °C) FMDV or VLP preparations in bicarbonate coating buffer and incubated overnight at 4 °C. The plates were washed three times with 300 µl per well wash buffer (0.01 M PBS, 0.15% polysorbate 20, 0.64 M NaCl and 0.0027 M KCl). After 1 h of blocking at 37 °C with 200 µl/well blocking buffer [40 mM Tris, pH 7.4, 0.2% (w/v) casein sodium salt, 0.05% (v/v) Triton X-100 and 4% sucrose], the plates were washed three times with 300 µl per well wash buffer, and Ab117 (1 µg ml^−1^ and 100 µl per well) in dilution buffer (200 mM PBS containing 0.05% polysorbate 80 and 0.1% BSA) was added to the plate. After another 1 h incubation at 37 °C and subsequent washing, an anti-Ruminant-PO conjugate (100 µl per well) in dilution buffer was added. After 1 h incubation at 37 °C, plates were washed three times with 300 µl per well wash buffer. Substrate (3,3′,5,5′-TMB) in urea hydrogen peroxide buffer was added, 100 µl per well, and incubated at room temperature in the dark for at least 15 min. The substrate reaction was stopped by adding 50 µl per well 2 M (4 N) sulphuric acid. The OD450 value of each well was read using a Tecan Sunrise spectrophotometer.

## Results

### Isolation and binding characterization of Ab117

A total of 143 paired heavy-and-light chain bovine antibody sequences were obtained, of which 50 were selected for recombinant expression providing a range of CDR-H3 sequence length and diversity.

**Fig. 1.**
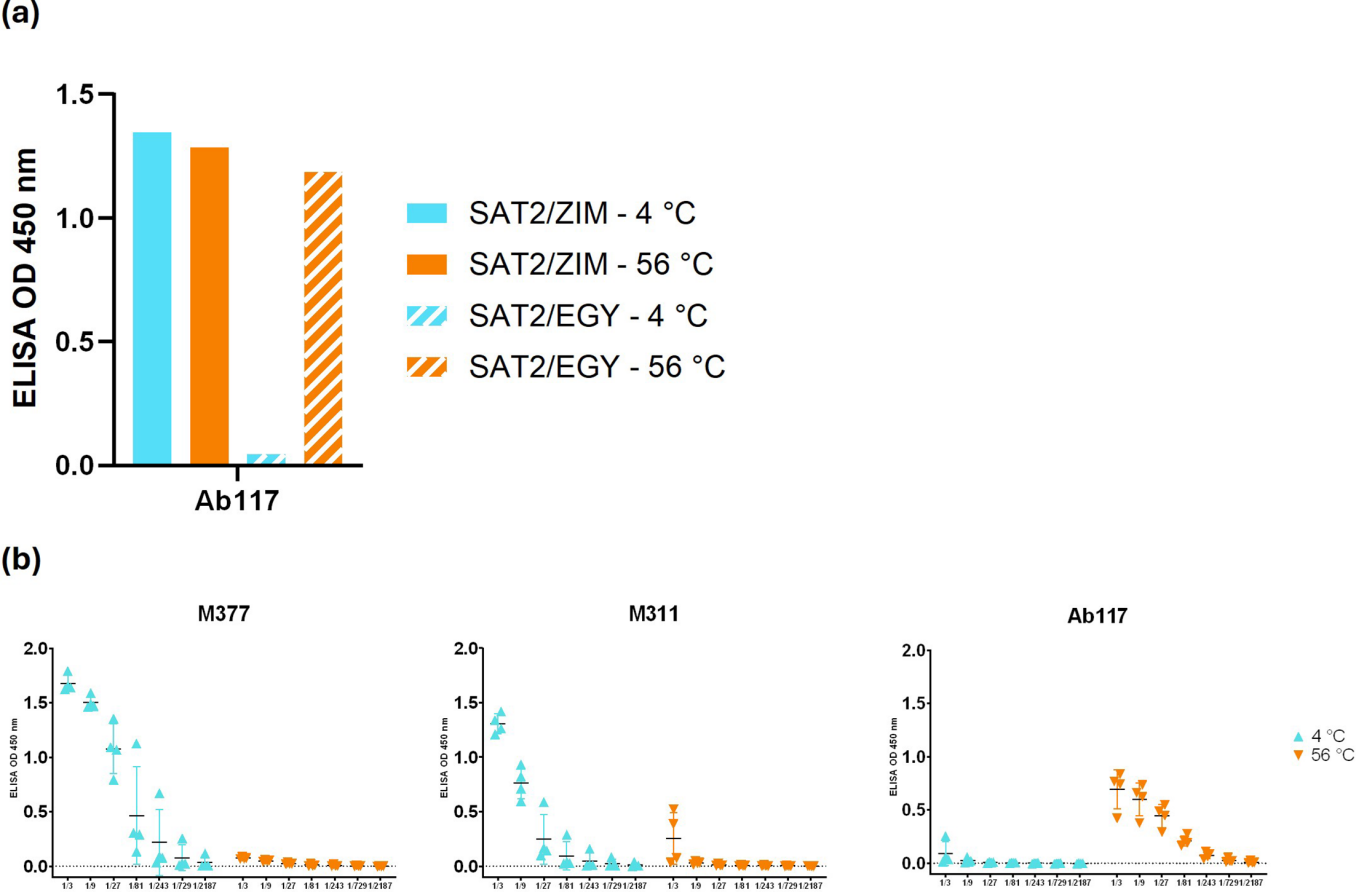
(**a**) Double sandwich homologous ELISA of Ab117 reactivity (the complex is not pre-made before application to the ELISA plate) with SAT2 topotypes. SAT2/ZIM/7/83 VLPs with S2093Y and SAT2/EGY/4/2012 inactivated virus. (**b**) Antigen titration ELISA (starting from 3.43 µg ml^−1^ as 1/3 dilution) utilizing M377, M311 and Ab117 as both capture and detection antibody for the SAT2/EGY/4/2012 inactivated virus without and with heat treatment (56 °C for 15 min) to disrupt the capsids.

We found that one of these antibodies had unusual properties detected by ELISA analysis. Ab117 (Table S2) did not exhibit binding to untreated SAT2/EGY inactivated virus, while it produced a signal to thermally disrupted viral particles and to intrinsically unstable SAT2/ZIM-S2093Y VLPs (Fig. 1a, b), which produced a signal with Ab117 regardless of heat treatment; this indicated that VLPs were dissociated prior to any application of heat treatment. Furthermore, immunoblot of linearized VLP serotypes showed that Ab117 accesses a non-linear epitope (Fig. S1). These data show that Ab117 could be useful in assessing disrupted FMDV antigens. Currently, only two VHHs (M377 and M311) are publicly available to detect FMDV antigens from the SAT2 serotype [16], with both VHHs exhibiting a preference for binding 146S antigen (Fig. 1b). However, M311 also produces meaningful signal with disrupted capsids, so it cannot be reliably used for SAT2 12S-/146S-specific determination, whilst M377 exhibits only very weak interactions with disrupted capsids. Ab117 showed a 100-fold enhanced sensitivity towards disrupted SAT2 capsids (down to 0.014 µg ml^−1^; 1/729 dilution), in contrast with 1.14 µg ml^−1^ (1/9 dilution) for M377 and M311 VHHs (Fig. 1b), demonstrating the effectiveness of Ab117 as a reagent for detection of disrupted SAT2 capsids in vaccine QC.

### Ab117 binds at the internal surface of dissociated VLP pentamers

**Fig. 2.**
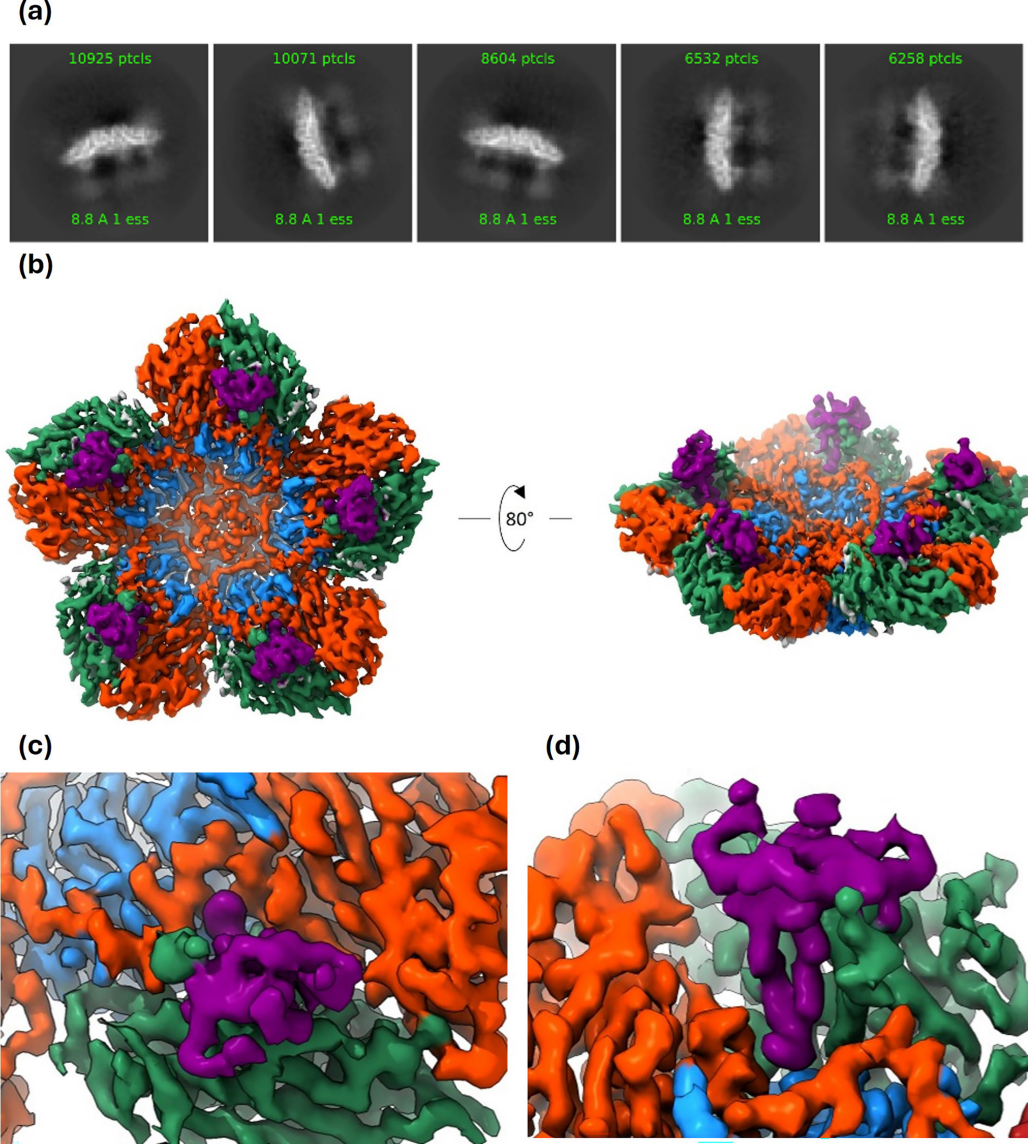
Fab117 binds to the interior surface of the dissociated SAT2/ZIM/7/83 pentamer. (**a**) Representative 2D class averages. (**b**) Final 3D reconstruction. (**c, d**) Close-up of the Fab117-SAT2/ZIM/7/83 complex density, viewed towards (**c**) the interior capsid surface and (**d**) from above the fivefold axis looking downward at the hydrophobic binding pocket. Density coloured by molecule: VP1 – blue, VP2 – turquoise, VP3 – orange and Fab117 CDR-H3 – purple.

The recombinant Fab fragment of Ab117 was produced by transient expression in Expi293 cells, and a structure of the complex of Fab117-SAT2/ZIM-S2093Y VLP-derived pentamer was determined by single-particle cryo-EM (Methods). From 110 210 classified particles, a final map to 3.2 Å resolution (Table 1, Figs 2 and S2) was reconstructed using C5 symmetry. Note that although there was a strong preference for edge-on orientations, the fivefold symmetry axes lying roughly in the plane of the grid produced complete reciprocal space sampling (Fig. 2a). The reconstruction showed pentamers decorated with small nodules on what would be the RNA-facing interior of an assembled capsid (Fig. 2a, b). Density (Fig. 2c, d) was sufficient to confidently model a pentamer of SAT2 and the distal part of the ultralong CDR-H3 of Fab 117 (Table 1).

### Fab117 structure

**Fig. 3.**
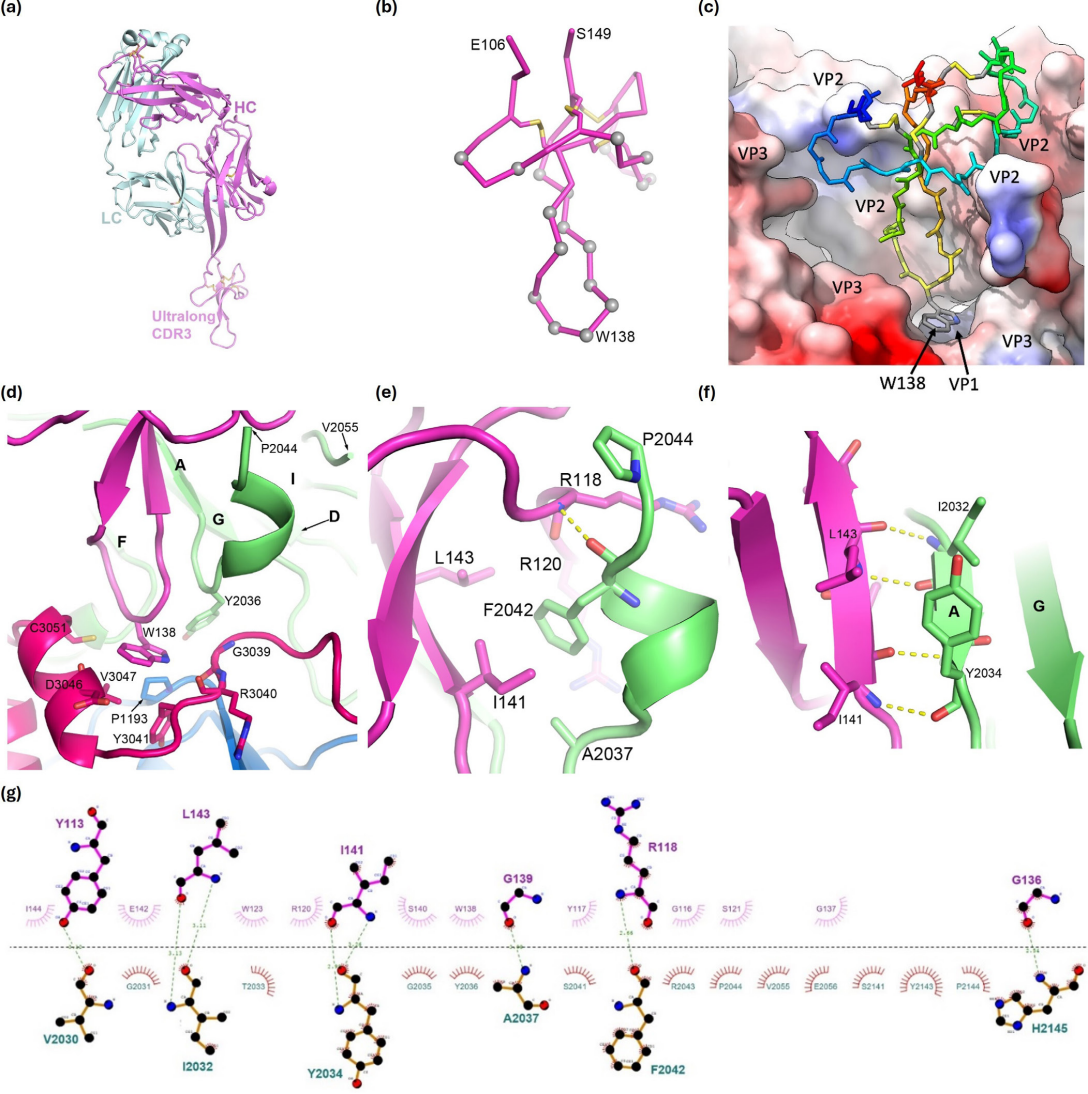
Details of Fab117 binding. For clarity, only interacting residues and bonds are shown. Residues are labelled with those from the virus given a preceding chain identifier for VP1, VP2 or VP3, respectively, such that they all have four digits. (**a**) AlphaFold model of Fab117 with HC in magenta and LC in cyan. (**b**) Cα trace of the ordered ‘knob’ domain of CDR-H3 in the SAT2 pentamer–Fab117 complex. Cαs of residues contacting the antigen (within 4.0 Å) are shown as grey spheres and disulphides as yellow sticks. The CDR-H3 is orientated as in (**a**). (**c**) Fab117 (rainbow backbone) engages the hydrophobic cleft of the SAT2 pentamer shown in surface representation and coloured by hydrophobicity. (**d**) The tip of the Fab117 CDR-H3 is shown in purple with W138 central and SAT2 residues within 4.0 Å of the latter represented as sticks. VP2 is coloured green, VP3 red and the antibody purple, with VP2 secondary structural elements labelled. (**e, f**) Zoomed perspective of the interactions between Fab117 and VP2. VP2 secondary structural elements are labelled. (**g**) LigPlot+v2.2.8 [38] of the interaction between the Fab117 CDR-H3 and interface residues of VP2 only (thus, for instance, the contacts of W138 to VP1 and VP3 are not shown).

The modelled Fab117 (Fig. 3a) showed that the ultralong CDR-H3 (residues E106-S149) largely conforms to the fold predicted by AlphaFold [32] (RMSD over 44 superimposed Cαs: 1.0 Å, Fig. S3), with an extended beta hairpin loop and a knob sub-domain characteristic of ultralong cattle CDR-H3s (Fig. 3b) [19]. Four disulphide bonds, between residues 109 and 131, 115 and 147, 119 and 130 and 125 and 146, stabilize the knob in a 1–6, 2–8, 3–5 and 4–7 configuration, and this sub-domain has a further extended hairpin loop of 12 residues projecting further outwards from the body of the Fab, at the tip of which is a tryptophan residue W138 (Fig. 3b). Only the knob and hairpin bearing the tryptophan residue are visible in the density; beyond this, the density rapidly fades away, although diffuse density in the 2D class averages suggests that the body of the Fab is positioned away from the pentamer and thus less constrained to C5 symmetry.

### Ab117 engages a hydrophobic epitope

Fab117 binds the internal surface of the SAT2 12S-dissociated pentamer at the quasi-threefold of the icosahedral protomer with W138 buried in a deep pocket (Fig. 2d), so that all the interactions between Fab117 and the pentamer lie within a protomeric subunit (Fig. 3). Fig. 3c shows that the knob mini-domain of Fab117 engages the SAT2/ZIM/7/83 protomeric subunit via a hydrophobic cleft dominantly comprising the quasi-threefold axis facing interior surface of VP2, with additional glancing contacts from VP3, and there is also a single-point contact with VP1 at the very base of the binding cleft. The interacting residues were identified using Proteins, Interfaces, Structures and Assemblies (PISA) [37] and LigPlot+ [38]. The structure of the dissociated pentamer differs somewhat from that of pentamers in the mature particle [39], largely due to a small rotation of VP3 [40]. The CDR-H3 W138 protrudes from the furthestmost tip, contributing 154 Å^2^ to the surface area buried on binding. It is stabilized by a stacking interaction with P1193, which is highly conserved across all serotypes. W138 and the preceding two glycine residues of Fab117 fit tightly within a pocket bordered by α_z_ and β_B-1_ (residues 3039–3051) of VP3 (pink), the β_A2_α_z_ turn (residues 2036 and 2142–2145) of VP2 (green) and floored by residues 1192–1193 of VP1 (for secondary structure nomenclature, see [12]). The total surface area buried by the CDR-H3 is 1292 Å^2^. The majority of these interactions are with VP2 (buried surface area 833 Å^2^) and much less with VP3 (266 Å^2^) and VP1 (193 Å^2^). Of the H-bond interactions between the Fab and the capsid, all are with protein main-chain atoms. The extended hairpin of the CDR-H3 knob is attached antiparallel to the VP2 N-terminal strand β_A_ (residues I2032–Y2034), which itself is pinned parallel to strand β_G_ of the VP2 beta-barrel core (Fig. 3d–f), whilst VP2 residues D2038–F2042 refold from a loop to a short helix making a main-chain interaction with Fab residue R118 and hydrophobic interactions from F2042 to Fab residues R120, I141 and L143 (Fig. 3g).

### Main-chain interactions confer cross-specificity of Ab117 to internal viral and VLP epitopes

**Fig. 4.**
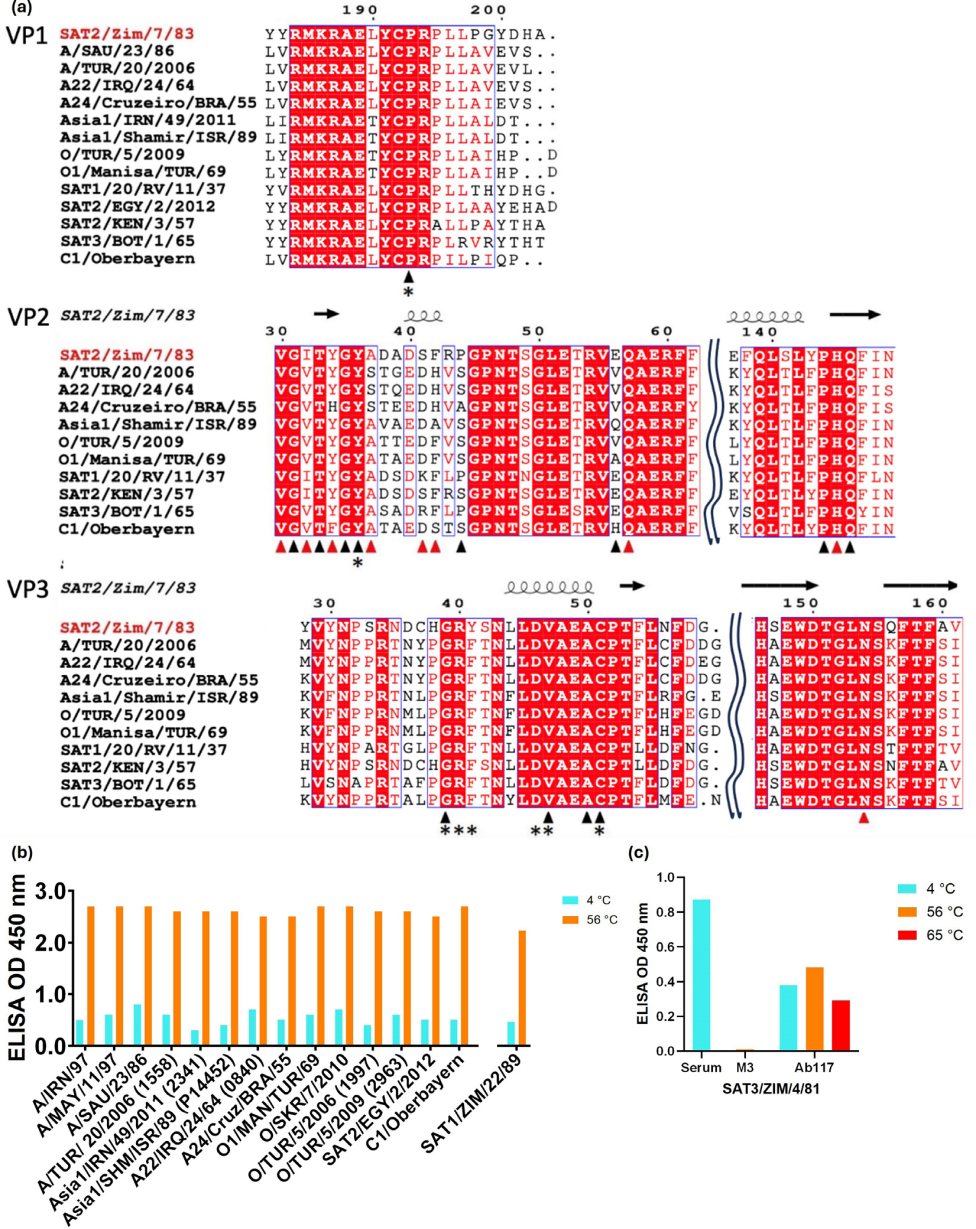
Ab117 cross-specificity. (**a**) Sequence alignment of the representative FMDV serotypes [28,30,52]. Residues with red background are conserved. Residues at the Fab117–SAT2/ZIM/7/83 interface are boxed. Black triangles indicate appreciable contribution to the interface, whilst red triangles indicate a hydrogen bond interaction (contributions determined using PISA). Residues directly making contact with W138 (≤4.0 Å) are indicated using an asterisk. The secondary structure of the SAT2/ZIM/7/83 proteins is indicated above the alignment. Alignment was performed using CLUSTALW [60,61], and the figure was generated using ESPript [41]. (**b**) Heterologous sandwich ELISA shows pan-specific binding of Ab117 to thermally disrupted virus. Integrin α_v_β_6_ was used as a universal capture for all intact viruses, whilst heated particles were trapped directly on the plates, with Ab117 applied for detection. SAT1/ZIM/22/89 inactivated virus was tested in an independent experiment. (**c**) Ab117 binding to SAT3/ZIM/4/81 [62] wild-type VLPs. Serum raised in animals infected with SAT3 was used as a positive control, whilst the lack of M3 binding to disrupted capsids showed the inability of this VHH to recognize SAT3-disrupted particles. Ab117 binds to untreated capsids, suggesting their instability. Heating at 56 °C increased the signal, consistent with increased disruption of particles, while heating at 65 °C exhibited a lower signal, which suggests the disruption of the conformational epitope recognized by Ab117 at higher temperatures. Cyan bars represent the signal using untreated capsids (4 °C), orange, and red bars represent the binding level using capsids heat treated for 15 min at 56 °C and 65 °C, respectively.

To assess the overall conservation of the epitope, we used ESPript [41] (Fig. 4a), which shows that the epitope residues, especially those lining the pocket occupied by W138, are highly conserved.

ELISAs using examples of FMDVs from all seven serotypes each with intact 146S particles (trapped with integrin) and dissociated 12S pentamers (coated directly on the plate) and detected with Ab117 showed pan-serotype binding to dissociated pentamers (Fig. 4b, c). 12S particles from all seven serotypes with multiple strains within serotypes O, A and Asia1 were detected. Wild-type SAT3 VLPs were also used to assess the ability of Ab117 to bind disrupted capsids from this serotype. Given the instability of these VLPs, Ab117 was able to bind both untreated and, to a higher extent, heated SAT3 VLPs (Fig. 4c), which was not the case of the M3 VHH used as a negative control. Heating of VLPs at 65 °C for 15 min is likely to disrupt conformational epitopes, leading to a decrease of Ab117 binding.

Additionally, O1M wild-type VLPs were used to assess the binding of Ab117 by ELISA and confirmed the binding of Ab117 to dissociated pentamers from VLPs as well as the virus (Fig. S4).

Altogether, these data confirm the ability of Ab117 to bind disrupted capsids from seven serotypes, and this is the first tool described to allow disrupted capsid SAT2 capsid quantification and disrupted SAT3 capsid binding.

## Discussion

FMDV probes have been largely mouse antibodies or VHH from llamas [16,42] and have only occasionally been generated in cattle [43]. These have been classified as recognizing 146S intact virus [16,44]/75S VLPs or, alternatively, 12S pentamers. Previously, all such reagents have been found to bind to the capsid exterior with a footprint contained either within a pentameric subunit [40,45, 46] or across a pentamer interface for those that distinguish intact from dissociated particles [16,40, 47]. As the exterior capsid surface is far more variable between serotypes than the interior surface [48], these reagents are generally serotype specific. In addition, many (particularly murine) anti-FMDV antibodies recognize linear epitopes (having been raised against peptides) [49] and thus would not be useful for QC of capsid assembly.

Ab117 is a so-called ultralong CDR-H3 bovine antibody, arising following immunization with dissociated capsids. The structure of Fab117 in complex with the pentamer of the serotype SAT2 shows that the antibody binds to the interior surface. Binding is mediated exclusively via the addition of ultralong CDR-H3 to the tertiary structure of VP2 with the tryptophan residue at its tip buried deep in a conserved hydrophobic pocket, which, based on its size (a tryptophan is similar in size to an RNA base) and hydrophobicity, we hypothesize that it might possibly be recognized by an RNA base during genome packaging. The insertion of Fab117 into this recess in the SAT2 pentamers provides another example of an ultralong antibody recognizing a deep epitope inaccessible to antibodies with shorter CDRs that tend to present a relatively flat paratope comprising both VH and VL domain CDRs, e.g. R50 (PDB ID: 7D3M) [22]. NC-Cow1 (PDB ID: 6OO0) [21] is another ultralong bovine antibody able to access epitopes invisible to canonical antibodies; although as the structure of the complex of antibody F145 with the FMDV serotype O/Tibet/99 (PDB ID: 7D3L) [22] has shown, this is not always the case for these antibodies. For Ab117, regions of the CDR-H3 zipper up to VP2 via secondary structure-like interactions. Thus, the ultralong CDR-H3 makes extensive sequence-independent main-chain interactions with the FMDV capsid proteins, and the epitope, as a whole, is highly conserved across FMDV serotypes, indicating that Ab117 would detect dissociated pentamers across FMDV serotypes. This was confirmed experimentally by ELISAs of dissociated capsids and demonstrates the utility of Ab117 for assessing the assembly state of recombinant capsids.

Intact FMDV antigen elicits a protective response, while dissociated antigen does not [50,51]. We propose Ab117 as a useful reagent for testing the integrity of vaccine antigen preparations.

## supplementary material

10.1099/jgv.0.002032 Uncited Fig. S1.

## Abbreviations

A22: foot-and-mouth disease virus serotype A22/IRQ/24/64
A1S: foot-and-mouth disease virus serotype Asia1/SHA/ISR/89
CDR: complementarity determining region
cryo-EM: cryogenic electron microscopy
DPBS: Dulbecco’s PBS
Fab: fragment antibody
FMD: foot-and-mouth disease
FMDV: foot-and-mouth disease virus
FSC: Fourier shell correlation
HC: heavy chain
LC: light chain
MWCO: molecular weight cut off
OD450: optical density at 450 nm
O1M: foot-and-mouth disease virus serotype O1/MAN/TUR/69
PISA: Proteins, Interfaces, Structures and Assemblies
QC: quality control
S2S: foot-and-mouth disease virus serotype SAT2/SAU/6/200
TMB: tetramethylbenzidine
VHH: single-domain heavy chain only antibody
VLPs: virus-like particles
VP: viral protein
VVs: vaccinia viruses
WBVR: Wageningen Bioveterinary Research
