# Supplementary material for: A broadly reactive ultralong bovine antibody that can determine the integrity of foot-and-mouth disease virus capsids
Source: J Gen Virol. 2024 Oct 18;105(10):002032. doi: 10.1099/jgv.0.002032 (PMC11488517; doi:10.1099/jgv.0.002032)
Supplement: Uncited Fig. S1. [file jgv-105-02032-s001.pdf]

A broadly reactive ultralong bovine antibody that can determine the integrity of foot-and-mouth disease virus capsids

Supplementary material

1.1 Author names

John D. Clarke [1,2,3,†] [https://orcid.org/0000-0001-7891-7626], Helen M.E. Duyvesteyn [1] [https://orcid.org/0000-0002-4641-5442], Eva Perez-Martin [2] [https://orcid.org/0000-0002-4355-4367], Undīne Latišenko [5] [https://orcid.org/0009-0005-8802-6479], Claudine Porta [1] [https://orcid.org/0000-0002-6788-4141], Abigail L. Hay [2] [https://orcid.org/0000-0002-8143-2109], Jingshan Ren [1] [https://orcid.org/0000-0003-4015-1404], Elizabeth E. Fry [1] [https://orcid.org/0000-0001-9754-5303], Erwin van den Born [5] [https://orcid.org/0000-0002-0341-2874], Bryan Charleston [2] [https://orcid.org/0000-0002-6952-9338], Marie Bonnet-Di Placido [2] [https://orcid.org/0000-0002-8299-5595], Raymond J. Owens [1,4,*] [https://orcid.org/0000-0002-3705-2993], David I. Stuart [1,3,6,*] [https://orcid.org/0000-0002-3426-4210], and John A. Hammond [2,*] [https://orcid.org/0000-0002-2213-3248].

1.2 Affiliation(s)

[1]The Division of Structural Biology, Nuffield Department of Medicine, The Centre for Human Genomics, University of Oxford, Oxford, OX3 7BN, United Kingdom

[2]The Pirbright Institute, Woking, GU24 0NF, United Kingdom

[3]Diamond Light Source, Didcot, OX11 0DE, United Kingdom

[4]Structural Biology, The Rosalind Franklin Institute, Didcot, OX11 0QX, United Kingdom

[5] MSD Animal Health, 5831 AN Boxmeer, The Netherlands

[6] Chinese Academy of Medical Sciences (CAMS) Oxford Institute (COI), University of Oxford, Oxford, OX3 7BN, United Kingdom

[†]*John D. Clarke current address: Structural Biology, The Rosalind Franklin Institute, Didcot, OX11 0QX, United Kingdom*

1.3 Corresponding author and email address

[*]Raymond J. Owens, email: raymond.owens@strubi.ox.ac.uk, David I. Stuart, email: dave.stuart@strubi.ox.ac.uk, John A. Hammond, email: john.hammond@pirbright.ac.uk

1.4 Keywords

FMDV; ultralong antibody; single particle analysis; pan-specific; vaccine quality assurance

1.5 Repositories:

The amino acid sequence of Antibody117 is provided in the supplementary material. EM maps and models for the SAT2/Zimbabwe/7/83 12S pentamers in complex with Fab117 knob mini-domains are deposited in the wwPDB and EMDB under accession codes, PDB: 9G6V, EMDB: EMD-51105.

Supplementary Table 1. Capture and Detection antibodies used in VHH double sandwich ELISA. Pirbright obtained all VHH (denoted as M3 or Mnnn) directly from WBVR. Ab117 was produced by The Immunological Toolbox (35). Detection antibodies are biotinylated capture antibodies.

Serotype	Particle	Antibody
A22/Iraq/24/64	75S	M702
A22/Iraq/24/64	12S	M3
Asia1/Shamir	75S	M332
Asia1/Shamir	12S	M3
O1/Manisa/Turkey/69	75S	M170
O1/Manisa/Turkey/69	12S	M3
SAT2/Zimbabwe/7/83	75S	M377/M311
SAT2/Zimbabwe/7/83	12S	Ab117

Supplementary Table 2. Amino acid sequences for Ab117 variable domains.

**VH:**
QVQLRESGPSLVKPSQTLSLTCTASGFSLSDKAVGWVRQAPGKALEWLGSVDSGGNTGYNPGLKSRLSITKDNSK SQVSLSVSSVTTEDSATYYCTTVLQQTTKKENNCPDGYSCGYRCRSGWGCSGDECCGRRGGGWGSIELIACCSST YIHEFHVDAWGQGLLVTVSS
**VL:**
QAVLTQPSSVSGSLGQRVSITCSGSSSNIGNSNVGWYQQVPGSGLTTIIYSSFSRPSGVPDRFSGSKSGNTATLTISS LQAEDEADYFCATYDSGVSIGVFGSGTTLTVL

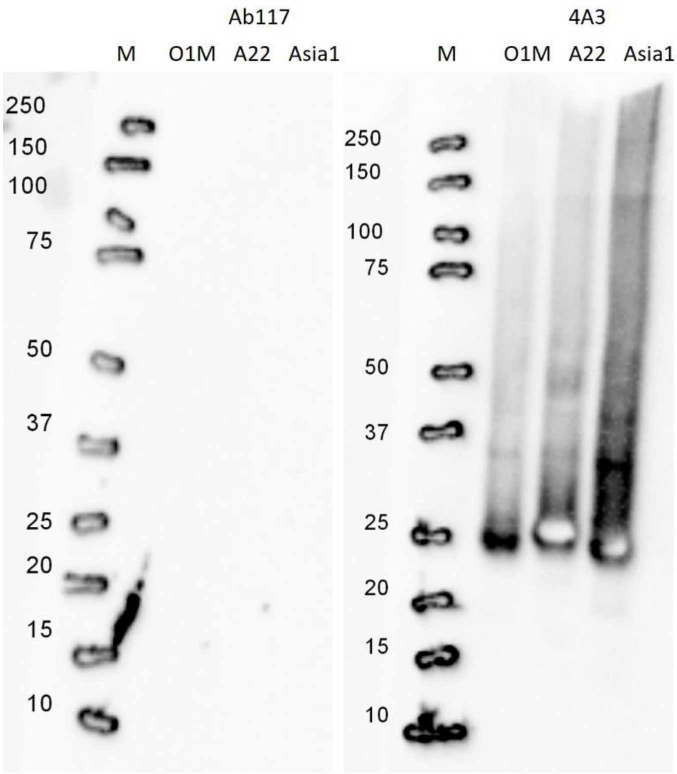

Supplementary Figure 1. Antibody 117 does not recognise a linear epitope of FMDV. VLPs were thermally disrupted to produce linear epitopes, and bound antibodies detected by a sheep anti-bovine-HRP antibody. Full protocol for this western blot is provided as follows in the supplemental material. Mouse anti-VP2 antibody 4A3 recognises a linear epitope (63). 'M' denotes marker lane.

Supplementary material and methods:

Western blotting was used to determine whether the Ab117 was capable of binding linearised epitopes. O1M, A22, and A1S VLPs were fully reduced using 10X NuPAGE reducing agent (Thermo Fisher Scientific) at 95 °C for 15 minutes and VP subunits separated by SDS-PAGE. Proteins were transferred to the western blot using the iBlot2 (Invitrogen). Blots were blocked with 5% milk (Marvel) in PBS-Tween (0.05% Tween) for two hours at room temperature. Primary antibody (Ab117) was diluted to 0.2 µg/ml in 1% milk PBST and incubated on the blot for one hour at room temperature. Secondary sheep anti-bovine-HRP (BioRad) was used at a 1/10,000 dilution in 1% milk and incubated on blot for one hour at room temperature. For mouse-anti-VP2 antibody 4A3, the concentration could not be determined as the antibody was unpurified, so a dilution of 1/5000 was used, and goat anti-mouse-HRP (BioRad) was used as the secondary antibody. Blots were washed three times for 5 minutes in PBST between each step. Blots were developed using ECL Prime (Amersham) according to the manufacturer's instructions and imaged on the BioRad ChemiDoc.

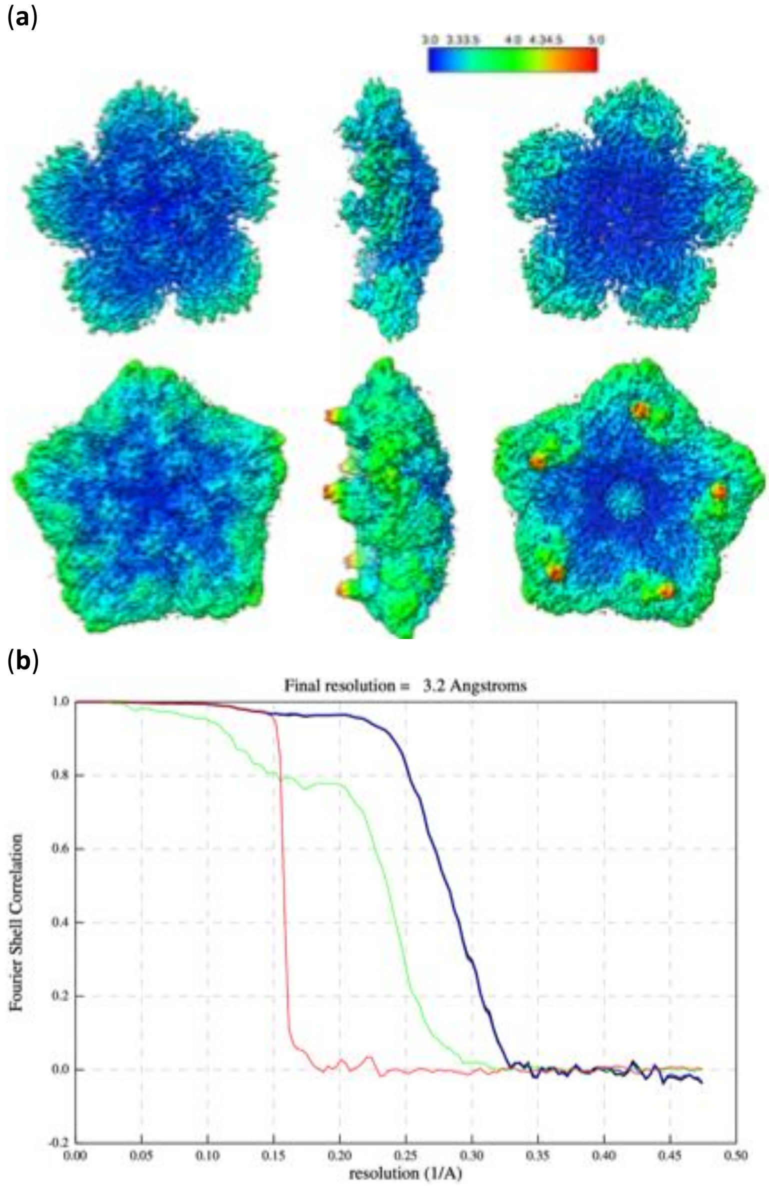

Supplementary Figure 2. Refinement of electron density map. (a) Representative map density contoured at 1.0 (top) and 0.2 (bottom) σ, coloured by determined local resolution. (b) Auto-tightened GSFSC plot indicating determined resolution. Red – Tight mask (2.1 Å), Green – Loose Mask (2.3 Å), Blue – No Mask (2.6 Å), Purple – Corrected (2.1 Å).

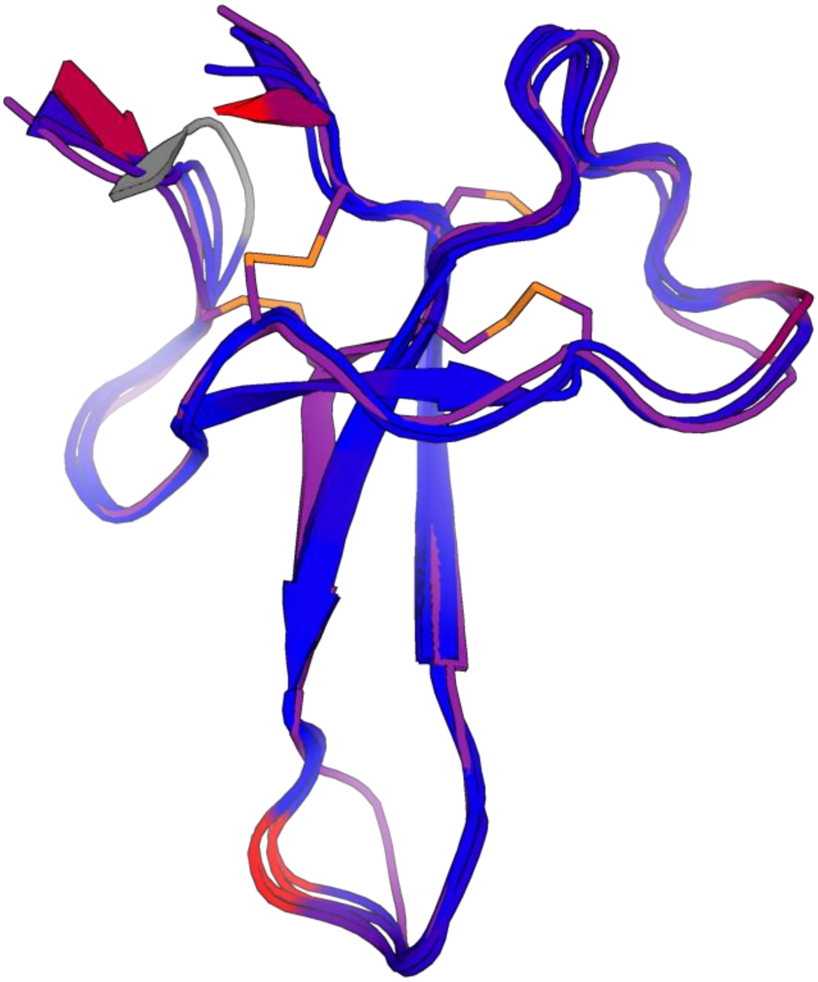

Supplementary Figure 3. Superposition of observed and predicted Ab117 CDR-H3 knob mini-domains in cartoon representation. Ab117 was modelled using AlphaFold (32) v2.0 multimer mode. RMSD represented by colouring of AlphaFold models main chain from 4.90 Å RMSD (red) to 0.15 Å RMSD (blue), as calculated using the PyMOL ColorByRMSD script (64). The short stretch coloured grey could not be aligned using ColorByRMSD. Observed Ab117 knob conformation coloured purple, and disulphide bridges indicated. Knob mini-domains orientated to match Figure 3b.

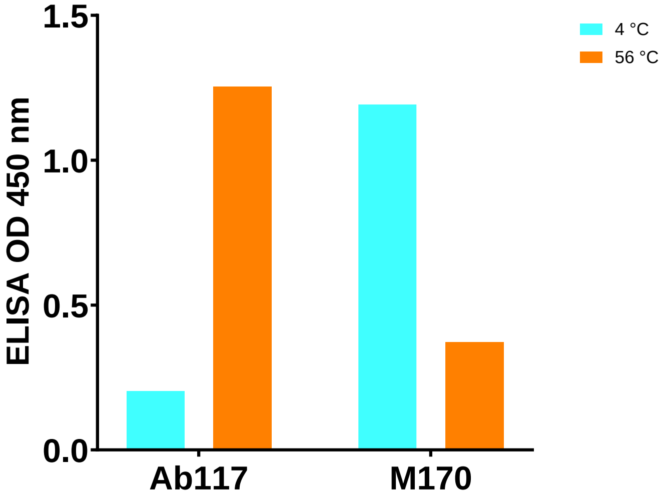

Supplementary Figure 4. Ab117 specifically binds to disrupted O1M wild type (non-stabilised) VLPs. Capsid disruption showed increased signal for Ab117, consistent with a decreased signal of M170 VHH binding, which is specific to intact O1M antigens.

Supplementary References

63. Asfor A, Howe N, Grazioli S, Berryman S, Parekh K, Wilsden G, et al. Detection of bovine antibodies against a conserved capsid epitope as the basis of a novel universal serological test for footand-mouth disease. Journal of Clinical Microbiology. 2020; 58(6): e01527-19.

64. S handilya S, Vertrees J, Holder T. ColorByRMSD. [Online]. [cited 2022 05 03]. Available from: https://pymolwiki.org/index.php/ColorByRMSD.
